# Functional proteomics can define prognosis and predict pathologic complete response in patients with breast cancer

**DOI:** 10.1186/1559-0275-8-11

**Published:** 2011-07-08

**Authors:** Ana M Gonzalez-Angulo, Bryan T Hennessy, Funda Meric-Bernstam, Aysegul Sahin, Wenbin Liu, Zhenlin Ju, Mark S Carey, Simen Myhre, Corey Speers, Lei Deng, Russell Broaddus, Ana Lluch, Sam Aparicio, Powel Brown, Lajos Pusztai, W Fraser Symmans, Jan Alsner, Jens Overgaard, Anne-Lise Borresen-Dale, Gabriel N Hortobagyi, Kevin R Coombes, Gordon B Mills

**Affiliations:** 1 Departments of Breast Medical Oncology and Systems Biology, The University of Texas MD Anderson Cancer Center, 1515 Holcombe Blvd, Houston, TX 77030, USA; 2 Departments of Gynecology Medical Oncology and Systems Biology, The University of Texas MD Anderson Cancer Center, 1515 Holcombe Blvd, Houston, TX 77030, USA; 3 Department of Surgical Oncology, The University of Texas MD Anderson Cancer Center, 1515 Holcombe Blvd, Houston, TX 77030, USA; 4 Department of Pathology, The University of Texas MD Anderson Cancer Center, 1515 Holcombe Blvd, Houston, TX 77030, USA; 5 Department of Bioinformatics, The University of Texas MD Anderson Cancer Center, 1515 Holcombe Blvd, Houston, TX 77030, USA; 6 Department of Bioinformatics, The University of Texas MD Anderson Cancer Center, 1515 Holcombe Blvd, Houston, TX 77030, USA; 7 Department of Systems Biology, The University of Texas MD Anderson Cancer Center, 1515 Holcombe Blvd, Houston, TX 77030, USA; 8 Department of Genetics, Institute for Cancer Research, The Norwegian Radium Hospital, and Faculty Division The Norwegian Radium Hospital, Faculty of Medicine, University of Oslo, Sognsvannsveien 20, Oslo 0027 Norway; 9 Lester and Sue Smith Breast Center, Baylor College of Medicine, 1 Baylor Plaza # Bcm600, Houston, TX 77030, USA; 10 Department of Pathology, The University of Texas MD Anderson Cancer Center, 1515 Holcombe Blvd, Houston, TX 77030, USA; 11 Department of Pathology, The University of Texas MD Anderson Cancer Center, 1515 Holcombe Blvd, Houston, TX 77030, USA; 12 Department of Hematology and Oncology, Hospital Clinico Universitario de Valencia, Avenida Blasco Ibáñez, 17, Valencia, 46010, Spain; 13 Molecular Oncology and Breast Cancer Program, University of British Columbia, 2211 Wesbrook Mall, Vancouver, British Columbia V6T 2B5, Canada; 14 Department of Cancer prevention, The University of Texas MD Anderson Cancer Center, 1515 Holcombe Blvd, Houston, TX 77030, USA; 15 Department of Breast Medical Oncology, The University of Texas MD Anderson Cancer Center, 1515 Holcombe Blvd, Houston, TX 77030, USA; 16 Department of Pathology, The University of Texas MD Anderson Cancer Center, 1515 Holcombe Blvd, Houston, TX 77030, USA; 17 Department of Experimental Clinical Oncology, Aarhus University Hospital, Nordre Ringgade 1, DK-8000, Aarhus, Denmark; 18 Department of Experimental Clinical Oncology, Aarhus University Hospital, Nordre Ringgade 1, DK-8000, Aarhus, Denmark; 19 Department of Genetics, Institute for Cancer Research, The Norwegian Radium Hospital, and Faculty Division The Norwegian Radium Hospital, Faculty of Medicine, University of Oslo, Sognsvannsveien 20, Oslo 0027 Norway; 20 Department of Breast Medical Oncology, The University of Texas MD Anderson Cancer Center, 1515 Holcombe Blvd, Houston, TX 77030, USA; 21 Department of Bioinformatics, The University of Texas MD Anderson Cancer Center, 1515 Holcombe Blvd, Houston, TX 77030, USA; 22 Department of Systems Biology, The University of Texas MD Anderson Cancer Center, 1515 Holcombe Blvd, Houston, TX 77030, USA

**Keywords:** Breast Cancer, Functional Proteomics, Prognosis, Prediction

## Abstract

**Purpose:**

To determine whether functional proteomics improves breast cancer classification and prognostication and can predict pathological complete response (pCR) in patients receiving neoadjuvant taxane and anthracycline-taxane-based systemic therapy (NST).

**Methods:**

Reverse phase protein array (RPPA) using 146 antibodies to proteins relevant to breast cancer was applied to three independent tumor sets. Supervised clustering to identify subgroups and prognosis in surgical excision specimens from a training set (n = 712) was validated on a test set (n = 168) in two cohorts of patients with primary breast cancer. A score was constructed using ordinal logistic regression to quantify the probability of recurrence in the training set and tested in the test set. The score was then evaluated on 132 FNA biopsies of patients treated with NST to determine ability to predict pCR.

**Results:**

Six breast cancer subgroups were identified by a 10-protein biomarker panel in the 712 tumor training set. They were associated with different recurrence-free survival (RFS) (log-rank p = 8.8 E-10). The structure and ability of the six subgroups to predict RFS was confirmed in the test set (log-rank p = 0.0013). A prognosis score constructed using the 10 proteins in the training set was associated with RFS in both training and test sets (p = 3.2E-13, for test set). There was a significant association between the prognostic score and likelihood of pCR to NST in the FNA set (p = 0.0021).

**Conclusion:**

We developed a 10-protein biomarker panel that classifies breast cancer into prognostic groups that may have potential utility in the management of patients who receive anthracycline-taxane-based NST.

## Introduction

To inform decisions about therapy, it is necessary to have a better understanding of the molecular mechanisms underlying the heterogeneity of breast cancer. Transcriptional profiling revealed that breast cancer represents at least six molecular subtypes associated with different clinical features [1-3]. However, comprehensive analysis of breast cancer transcriptomes does not capture all levels of biological complexity; important additional information may reside in the proteome [4-7].

Proteins are the direct effectors of cellular function. Protein levels and function depend on translation as well as on post-translational modifications [6], which influence protein stability and activity [7]. Although many proteins have been studied as prognostic and predictive factors in breast cancer, only three alter current practice: estrogen receptor (ER), progesterone receptor (PR) and HER2. Thus, a systematic study of expression and activation of multiple proteins and signaling pathways may facilitate more accurate classification and prediction in breast cancer.

Neoadjuvant systemic therapy (NST) allows for in vivo assessment of chemosensitivity. Attaining a pathologic complete response (pCR) following NST provides a surrogate marker for improved long-term outcome. Conversely, patients with residual breast cancer after NST are at increased risk for recurrence and may have therapy-resistant disease [8-12].

The objective of this study was to apply functional proteomics to breast cancer classification and prognosis, and to develop a predictor of pCR in a group of primary tumor samples obtained by fine needle aspirations (FNA) from patients who subsequently received NST.

## Material and Methods

### Tumor tissues

Three sets of frozen breast cancer tissues were used: Training set (n = 712) was collected at M. D. Anderson Cancer Center (MDACC), Hospital Clinico Universitario de Valencia, Spain, University of British Columbia, Vancouver, BC, and Baylor College of Medicine, Houston, TX. Complete clinical information was available for 541 patients. Test set (n = 168) was obtained from an independent group of patients enrolled in the Danish DBCG 82 b and c breast cancer studies [13,14]. All tumors in the training and test sets were collected by excision during their primary surgery. Tumor content was verified by histopathology. The third set consisted of 256 FNAs obtained from primary breast cancers prior to NST of which 132 belonged to patients who subsequently received uniform taxane and anthracycline-based NST at MDACC (12 cycles of weekly paclitaxel or 4 cycles of every 3-week docetaxel, followed by 4 cycles of FAC or FEC100). All tissues were collected under Institutional Review Board-approved laboratory protocols.

Tumors were characterized for ER and PR status by immunohistochemistry (IHC), ligand-binding dextran-coated charcoal assay or reverse phase protein lysate array (RPPA). ER/PR positivity was designated when nuclear staining occurred in ≥10% of tumor cells, with ligand binding of ≥ 10 fmol/mg, or with a log2 mean centered cutoff of -1.48(ER) or +0.52(PR) by RPPA. Hormone receptor (HR) positivity was designated when either ER or PR was positive. HER2 status was assessed by IHC, fluorescent in situ hybridization (FISH) or RPPA. HER2 positivity was designated when 3+ membranous staining occurred in ≥10% of tumor cells, with a HER2/CEP17 ratio of > 2.0 or with a log2 mean centered cutoff of +0.82 by RPPA [15].

### Reverse phase protein lysate microarray (RPPA)

RPPA was completed independently and at different time points for training and tests sets using individual arrays. Protein was extracted from human tumors and RPPA was performed as described previously [16-19]. Lysis buffer was used to lyse frozen tumors by homogenization (excised tumors) or sonication (FNAs). Tumor lysates were normalized to 1 μg/μL concentration as assessed by bicinchoninic acid assay (BCA) and boiled with 1% SDS. Supernatants were manually diluted in five-fold serial dilutions with lysis buffer. An Aushon Biosystems 2470 arrayer (Burlington, MA) created 1,056 sample arrays on nitrocellulose-coated FAST slides (Schleicher & Schuell BioScience, Inc.). Slides were probed with 146 validated primary antibodies (Additional File 1, Table S1) and signal amplified using a DakoCytomation-catalyzed system. Secondary antibodies were used as a starting point for amplification. Slides were scanned, analyzed, and quantified using Microvigene software (VigeneTech Inc., Carlisle, MA) to generate spot signal intensities, which were processed by the R package SuperCurve (version 1.01) [18], available at "http://bioinformatics.mdanderson.org/OOMPA". A fitted curve ("supercurve") was plotted with the signal intensities on the Y-axis and the relative log2 concentration of each protein on the X-axis using the non-parametric, monotone increasing B-spline model [18]. Protein concentrations were derived from the supercurve for each lysate by curve-fitting and normalized by median polish. Protein measurements were corrected for loading as described [15-17,19]. For the selection of the 146 antibody set, we focused on markers currently used for breast cancer classification due to their value in treatment decisions (ER, PR, HER2). We then added additional antibodies to targets implicated in breast cancer pathophysiology, followed by antibodies to targets implicated in the pathophysiology of other cancer lineages. Final selection of antibodies was also driven by the availability of their high quality that could pass a strict validation process as previously described [20].

### Statistical Methods

Detailed statistical methods are described in Additional File 2.

### Identification of Prognostic Groups

To develop a set of markers for breast cancer classification and outcomes prediction, we used a hypothesis-driven approach, selecting markers according to their functional assignments and subsequently performing supervised proteomic clustering analysis to optimize the selection of groups with the most distinct recurrence-free survival (RFS) outcomes. We hypothesized that three functions would strongly affect the behavior and therapy responsiveness in breast cancer: ER function, grade/proliferation, and receptor tyrosine kinase activity. From the initial 146 antibodies, we selected markers within these three functional categories. We tested multiple combinations requiring that a minimum of one marker per functional category remain in each model. Unsupervised clustering analysis, using the uncentered correlation distance metric [21] and Ward's linkage rule [22], was applied to the training set to define groups and allow correlation with previously defined breast cancer subtypes. We then visualized the RFS curves to select the marker set that was associated with the clearest differences in RFS between the groups identified in the training set. Because of multiple testing and the possibility of false discovery, this model was locked and then applied to an independent test set to which the statistical analysis team was kept blinded. The selected protein groups were as follows: ER function (ER, ERpS118, ERpS167, PR, AR, EIG121, Bcl2, GATA3, IGF1R, and IGFBP2), grade/proliferation (CCNB1, CCND1, CCNE1, CCNE2, and PCNA), and receptor tyrosine kinase activity (cKit, EGFR, EGFRp1045, EGFRp922, HER2, HER2p1248, FGFR1, FGFR2, IGF1R, IGFRpY1135/Y1136).

RFS was estimated according to the Kaplan-Meier method and compared between groups using the log-rank statistic. Cox proportional Hazard Models were fitted using proteomic subgroups, selected markers and clinical variables.

### Decision trees

We constructed a statistical model to predict the classes discovered by hierarchical clustering using a binary decision tree with a logistic regression model at each node. The split at each node was a union of two of the classes. Protein-by-protein two-sample t-tests between the two halves of the split were computed. The proteins were ordered by p-value and then added one at a time into a logistic regression model until the desired prediction accuracy was achieved. In order to avoid overfitting data, a default precision accuracy of 95% was set for each node. Finally, the Akaike Information Criterion (AIC) was used to eliminate redundant terms from the logistic regression model [23].

### Validation of Prognostic Groups for RFS

The coefficients of the model, which used logistic regression at each node of a decision tree to place samples in one of six classes (or prognostic groups) were finalized and locked. An implementation of the model was provided to an independent analyst, along with the class predictions. The independent analyst was provided with the unblinded clinical data after implementation of the model. Cox proportional hazards models were then constructed using the predicted classes as covariates to test their association with RFS.

### Validation of Prognostic Groups for pCR

We applied the algorithm to the last sample set (132 FNAs) and correlated the groups with response to NST. We clustered the samples as above and compared these clusters to the class labels predicted by the decision tree model with Cohen's kappa statistic [24,25]. Using the predicted prognostic groups, we developed a Bayesian model to estimate the posterior probability of pCR in each group. We modeled the pCR rates as coming from a beta-binomial distribution [26].

### Development of a Prognostic Score and its Application to Prediction of pCR

We next converted the six prognostic groups into a continuous prognostic score (PS) by fitting an ordinal regression model on the training set [27]. PS is a weighted linear combination of the relative protein concentration of the markers:

PS = -0.2841*ER - 1.3038*PR + 0.0826*Bcl2 -0.6876*GATA3 + 0.5169*CCNB1 +

0.1000*CCNE1 + 0.4321*EGFR + 0.5564*HER2 + 0.8284*HER2p1248 +

0.2424*EIG121.

We used this formula to compute PS on the test set; PS was associated with RFS estimates by the Cox proportional hazards model. We also used the same formula to compute PS on the NST treated FNA set. We fitted a logistic regression model using the NST response as the binary response variable (pCR vs. residual disease) and PS as a predictor. The prediction of response was evaluated by a receiver operating characteristics (ROC) curve.

### Models for Recurrence-Free Survival and Likelihood of Pathologic Complete Response

A Cox proportional hazards model to estimate association with RFS was fit using each of the following covariates: prognostic group, tumor size, histologic grade, node status, each of the 10 protein markers, and PS. Using the same covariates, a logistic regression model was fit to estimate the association of each covariate with pCR. Stepwise multivariate model selection [28,29] was used to determine the combination of covariates for the multivariate models.

All statistical analysis was performed in R 2.8.1. (R Development Core Team (2008). R: A language and environment for statistical computing (R Foundation for Statistical Computing, Vienna, Austria). http://www.R-project.org.

## Results

### Unsupervised Proteomic Clustering

Table 1 summarizes the clinical characteristics of each set. Training set (n = 712) was analyzed for 146 proteins (Additional File 1, Table S1) using RPPA. Proteins were chosen based on a literature search of important targets and proteomic processes in breast cancer for which robust antibodies binding to a single or dominant band on western blotting could be identified and validated for RPPA as described [1-3,30-32]. Unsupervised clustering of the proteomic profiles is shown in Additional file 1: Figure S1. The 146 proteins stratified breast cancers into six major groups with different RFS outcomes (Additional file 1: Figure S2). The six groups included a predominantly HER2-positive group, a HR-negative and HER2-negative (triple receptor-negative) group with poor outcomes, a HR-positive group with a good outcome and three groups with intermediate outcome: an HR group with overexpression of proteins including cyclins B1 and E1 as well as components of the protein synthesis machinery including phosphorylated S6 ribosomal protein and 4EBP1, a group with overexpression of stromal markers including collagen VI, CD31 and caveolin1, and a group defined by up-regulation of a large number of proteins and phospho-proteins in several mechanistic pathways.

**Table 1:** Clinical characteristics of all sets

Characteristic	Training (n = 712)	Test (n = 168)	FNA (n = 256)	FNA subgroup (n = 132)
**Age**				
Median	62	56	50	50
Range	23-89	30-69	23-85	23-77
**T stage**	(n = 542)	(n = 166)	(n = 255)	(n = 132)
Tis	6	0	5	0
T1	165	49	22	14
T2	268	97	135	76
T3	37	20	42	24
T4	66	0	51	18
**N stage**	(n = 541)	(n = 166)	(N = 255)	(n = 132)
N0	280	0	102	47
N1	198	11	84	52
N2	39	75	15	13
N3	24	80	54	20
**Stage**	(n = 541)	(n = 166)	(n = 254)	(n = 132)
0	6	0	2	0
I	105	1	8	4
II	315	83	141	79
III	94	82	86	49
IV	21	0	18	0
**Histology**	(n = 576)	(n = 166)	(n = 255)	(n = 132)
Ductal	446	132	212	113
Other	130	34	43	19
**Grade**	(n = 457)	(n = 132)	(n = 251)	(n = 132)
1	65	29	12	8
2	149	69	72	39
3	243	34	167	85
**Estrogen Receptor Status**	(n = 709)	(n = 165)	(n = 255)	(n = 132)
Positive	447	126	149	79
Negative	262	39	106	53
**Progesterone Receptor Status**	(n = 709)	(n = 168)	(n = 255)	(n = 132)
Positive	336	82	108	56
Negative	373	86	147	76
**HER2 Status**	(n = 709)	(n = 128)	(n = 254)	(n = 132)
Positive	142	18	53	121
Negative	567	110	201	11
**Clinical Subtype**	(n = 709)	(n = 128)	(n = 254)	(n = 132)
Hormone receptor-positive	383	106	139	80
HER2-positive	142	40	53	11
Triple receptor-negative	184	22	62	41
**Systemic Treatment**	(n = 598)	(n = 168)	(n = 255)	(n = 132)
Adjuvant hormonal therapy	341	97	136	78
(Neo)Adjuvant chemotherapy	188	71	253	132
CMF-based	188	71	0	0
Anthracycline-based	0	0	21	0
Taxane-based	0	0	14	0
Anthracycline and Taxane-based	0	0	184	132
Trastuzumab-based	0	0	34	0
None	111	0	2	0

Note that numbers may not add up to the total in each category due to missing data. Tumors are assigned to the HR-positive group only if they are HER2-negative; tumors that are HER2-positive and HR-positive are classified in the HER2-positive group. FNA: Fine needle aspirates.

### Supervised Proteomic Clustering

**Figure 1:**
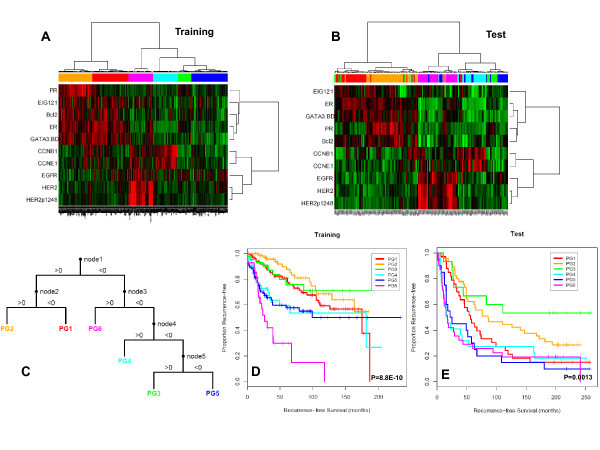
**Supervised clustering of breast cancers with quantification data for 10 proteins derived using reverse phase protein arrays**. The 712 breast tumor samples (Training set, **1A**) were clustered with the 10 markers using an "uncentered correlation" distance metric along with the Ward linkage rule. This analysis yielded six subgroups (BG1-6). The 168 breast tumor samples (Test set, **1B**) were subgrouped into one of 6 groups (PG1-6) using the decision tree (**1C**) that was derived from the training set. Patients in the six subgroups differed significantly in their recurrence-free survival in both training (**1D**) and test (**1E**) sets.

The hypothesis-driven approach described in Methods was applied to the training set and identified 10 markers in three functional groups known to be important to breast cancer behavior: ER function (ER, PR, Bcl2, GATA3, EIG121), tyrosine kinase receptor function (EGFR, HER2, HER2p1248), and cell proliferation (CCNB1, CCNE1). These markers separated the breast cancers into six subgroups (PG1 to 6) with markedly different RFS outcomes, (Log-rank p = 8.8 E-10), (Figures 1A and 1D). A decision tree model was developed (Figure 1C) that recovered the six subgroups of breast tumors identified by clustering with the 10 markers with an overall accuracy of 89%. Full description of the model is presented in Additional File 3. We then confirmed the presence of the six subgroups as well as their RFS in an independent test set, (Log-rank p = 0.0013), (Figures 1B and 1E). Table 2 summarizes the 5-year RFS estimates for each of the prognostic groups in the training and test sets.

**Table 2:** Five-year DFS estimates for each of the prognostic groups in both the training and test sets

5-year Recurrence-Free Survival Estimates Training Set Median follow-up 42.23 months (1.45-246.40 months)					
	**No. at Risk**	**No. of Events**	**5-Year Estimate**	**95% Confidence Interval**	**P-Value**

All	446	106	0.699	(0.65, 0.751)	
Prognostic Group 1	108	17	0.809	(0.730, 0.896)	
Prognostic Group 2	84	7	0.876	(0.793, 0.968)	
Prognostic Group 3	44	8	0.758	(0.620, 0.926)	
Prognostic Group 4	73	22	0.595	(0.464, 0.763)	
Prognostic Group 5	109	36	0.576	(0.472, 0.703)	
Prognostic Group 6	28	16	0.299	(0.152, 0.589)	8.88E-10

**5-year Recurrence-Free Survival Estimates Test Set** Median follow-up 217 months (180-259 months)					

	**No. at Risk**	**No. of Events**	**5-Year Estimate**	**95% Confidence Interval**	**P-Value**

All	166	92	0.446	(0.376, 0.528)	
Prognostic Group 1	33	18	0.455	(0.313, 0.661)	
Prognostic Group 2	45	17	0.622	(0.496, 0.781)	
Prognostic Group 3	15	5	0.667	(0.466, 0.953)	
Prognostic Group 4	22	16	0.273	(0.138, 0.540)	
Prognostic Group 5	20	14	0.300	(0.154, 0.586)	
Prognostic Group 6	31	22	0.290	(0.167, 0.503)	0.0013

**Figure 2:**
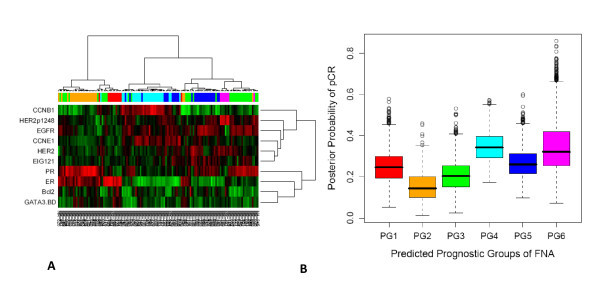
**The 132 fine needle aspirates from patients who received anthracycline and taxane-based neoadjuvant systemic therapy were subgrouped into one of the 6 groups using the decision tree from the training set**. Six true patient groups were obtained (**2A**), Cohen's kappa score = 0.66. Beta-binomial distribution and computed joint posterior probabilities were used to evaluate the association of the prognostic groups with pCR, the posterior distribution estimates of pCR by prognostic group are shown in **2B**.

We applied this classification approach to 256 FNAs from MDACC. In order to confirm that the same clusters were present, we compared the patient groups obtained by direct hierarchical clustering of the 256 FNA samples to the prognostic groups predicted in the FNA samples by the decision tree model derived from the training set (Cohen's κ = 0.70, p < 1E-20). The decision tree predictions were also applied to the subset of 132 FNAs from patients who received uniform anthracycline and taxane-based NST, and the same six clusters were found (Cohen's κ = 0.66, p value < 1E-20, Figure 2A). The association between pCR rates and the (predicted) prognostic groups did not quite reach statistical significance (χ^2 ^= 10.3076 on 5 degrees of freedom; p = 0.067). However, a Bayesian analysis of the pCR rates indicated that there was at least a 70% posterior probability that groups PG2 and PG3 have pCR rates at least 5% lower than those in PG4 or PG6 (Figure 2B).

### Prognostic Score Predicts pCR

**Figure 3:**
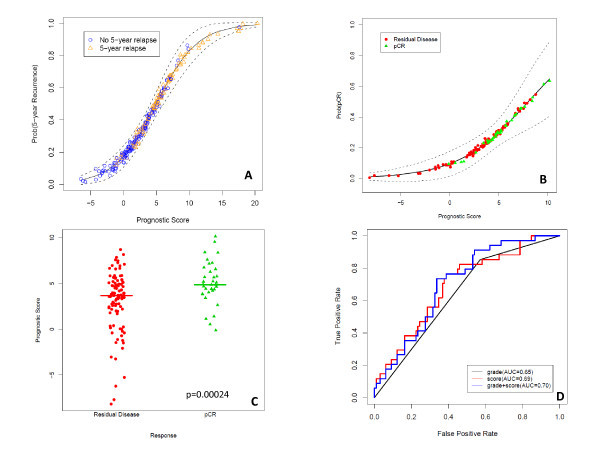
**A ten-protein prognosis score by ordinal regression modeling was derived from the training set**. **3A**. Probability of recurrence as a continuous function of the score. The rug plot shows the prognosis score for individual patients in the study. Dashed curves indicate the 95 percent confidence intervals. **3B**. Probability of pCR as a function of the prognostic score. **3C**. Stripcharts showing the level of prognostic score by response to anthracycline and taxane-based neoadjuvant systemic therapy. **3D**. Receiver operating characteristics curves for the performance of the prediction of pCR versus residual disease by the logistic model using the prognostic score. AUC: area under the curve.

As described in Methods, we computed a continuous prognostic score (PS) based on the grouping defined in the training set. A Cox proportional hazards model on the training set (CoxTrain) using PS to predict RFS was significant (Wald test; coefficient = 0.128, p = 3.2E-13). A second Cox model, fit on the test set (CoxTest), was also significant (Wald test; coefficient = 0.084, p = 1.1E-05) (Figure 3A). Of 132 patients who received anthracycline-taxane-based NST, 32 (24%) had a pCR. We computed the prognostic score PS for each FNA sample; the values ranged from -8.16 to 10.16. A logistic regression model showed that PS was also significantly associated with pCR (p = 0.0021, Figure 3B). Further, an unequal variance t-test comparing the prognostic scores between patients with pCR and residual disease also revealed a significant difference between mean scores (p = 0.00024 Figure 3C). The area under the curve (AUC) in a ROC curve analysis was 0.7 with a specificity of 98% and a negative predictive value of 76% (Figure 3D).

### Models for Recurrence-Free Survival and Likelihood of Pathologic Complete Response

Univariate models for RFS (Cox proportional hazards on the test set; CoxTest) and pCR (logistic regression on the uniformly treated FNA dataset; LR-FNA) are summarized in Table 3. All clinical and molecular variables, except for EGFR, were significantly associated with RFS. The addition of the prognostic score to the model with clinical covariates reduced the residual deviance with a X^2^_1 _= 2.96, p = 0.09. Stepwise model selection using AIC retained all clinical covariates and the prognostic score for the final model:

**Table 3:** Models for Recurrence-Free Survival and likelihood of pathological complete response

	RFS			pCR		
**Univariate Models**						

**Variable**	**Hazard Ratio**	**95% CI**	**Log-rank P-value**	**Odds Ratio**	**95% CI**	**Wald's P-Value**

Prognostic Group 1	1.59	(.87, 2.90)		3.54	(.06, 28.14)	
Prognostic Group 2	1.00	(1.0, 1.0)		1.00		
Prognostic Group 3	1.15	(.51, 2.60)		2.16	(.32, 17.82)	
Prognostic Group 4	3.12	(1.64, 5.90)		7.19	(1.77, 48.89)	
Prognostic Group 5	3.01	(1.67, 5.41)		4.24	(.90, 30.76)	
Prognostic Group 6	7.00	(3.53, 13.86)	<.0001	11.50	(1.40, 123.05)	.0519
Tumor size (</ = 2 cm vs. > 2 cm)	1.85	(1.16, 2.96)	.0094	1.30	(.56, 2.94)	.5364
Node status (positive vs. negative)	2.93	(1.99,4.29)	<.0001	1.11	(.50, 2.56)	.7981
Histologic grade (1 and 2 vs. 3)	3.70	(2.45, 5.60)	<.0001	4.35	(1.67, 13.62)	.0052
ER	0.82	(.76, .88)	<.0001	.73	(.56, .93)	.0180
PR	0.75	(.66, .85)	<.0001	.67	(.45, .91)	.0235
Bcl2	0.75	(.65, .86)	<.0001	.63	(.39, .96)	.0435
GATA3	0.77	(.66, .90)	.0010	.58	(.33, .95)	.0411
CCNB1	1.23	(1.12, 1.36)	<.0001	1.32	(1.00, 1.76)	.0449
CCNE1	1.40	(1.11, 1.76)	.0039	2.52	(1.32, 5.05)	.0062
EGFR	1.04	(.81,1.36)	.7437	1.54	(.90, 2.88)	.1333
HER2	1.21	(1.08, 1.36)	.0015	1.37	(.72, 2.57)	.3253
HER2p1248	1.18	(1.11, 1.26)	<.0001	1.09	(.74, 1.56)	.6528
EIG121	0.389	(.29, .52)	<.0001	.53	(.26, 1.05)	.0712
Prognostic score (continuous)	1.14	(1.10, 1.18)	<.0001	1.32	(1.12, 1.61)	.0021

**Multivariate Models**						

**Variable**	**Hazard Ratio**	**95% CI**	**Log-rank P-value**	**Odds Ratio**	**95% CI**	**Wald's P-Value**

**Clinical Characteristics Model**			1.87E-12*			.021*
Size	1.63	(.94, 2.85)	.0836	1.10	(.45, 2.63)	.8237
Node	3.90	(2.25, 6.75)	<.0001	1.07	(.56, 2.58)	.8732
Grade	2.75	(1.55, 4.85)	.0005	4.29	(1.64, 13.51)	.0057
**Clinical Model + Prognostic Score**			2.48E-12*			.004*
Size	1.51	(.86, 2.65)	.1489	1.18	(.47, 2.88)	.7192
Node	3.83	(2.22, 6.61)	<.0001	1.02	(.42, 2.51)	.9657
Grade	2.23	(1.21, 4.13)	.0106	2.41	(.80, 8.27)	.1332
Prognostic score	1.07	(.99, 1.16)	.0895	1.24	(1.03, 1.52)	.0327
**Tumor Grade + Prognostic Score**						.01*
Grade				2.46	(.83, 8.40)	.1198
Prognostic score				1.23	(1.03, 1.51)	.0283

RFS: Recurrence-free survival; pCR; pathologic complete response. * X^2 ^test

*log(h(t)/h_0_(t)) = 0.414Size + 1.34Node + 0.803Grade + 0.070PrognosticScore*.

For response (pCR vs. residual disease), grade was the only clinical covariate significantly associated with response. All protein markers except EGFR, HER2, pHER21248 and EIG121 were significantly associated with response. The addition of the prognostic score to grade reduced residual deviance with a X^2^_1 _= 5.39, p = 0.02. Stepwise model selection using AIC showed that both grade and prognostic score were retained in the final model:

*logit(pCR) = -2.61 + 0.902Grade + 0.2210PrognosticScore*.

We compared ROC curves for predicting pCR by the prognostic scores and the stepwise selected model and found that AUC, as well as the specificity and negative predictive values were the same (0.7, 98% and 76% respectively), suggesting that the prognostic score may be a more powerful predictor than clinical information.

## Discussion

We have identified and validated a 10-protein panel that accurately and reproducibly classifies patients with breast cancer into six subgroups with significantly different 5-year RFS times. These six groups included two HR positive groups differentiated primarily by PR levels with the PR high group having the best outcome, a HER2, pHER2 and EGFR positive group with the worst outcome (pre-trastuzumab treatment) and three triple negative groups, one with high cyclins and two groups without well defined selectors. Further, in an independent set of FNAs from patients who underwent NST, we were able to reproduce this classification and to use it to predict response to neoadjuvant anthracycline and taxane-based therapy. Further, in three independent sets, the 10-protein signature had a higher predictive value than clinical variables including tumor size, nodal status and grade in Cox models for RFS and in a logistic regression model to predict pCR.

Several studies using transcriptional profiling have classified breast cancer into different subtypes with implications in patient prognosis [1,30-32], frequency of genomic alterations [33,34], and therapy response [31,35,36]. Since proteins are the immediate effectors of cellular behavior, interrogation of the functional proteome is likely to complement data derived from transcriptional profiling. Thus, the integrated study of the expression and activation of multiple proteins and signaling pathways has the potential to provide powerful classifiers and predictors in breast cancer. As protein levels and function depend not only on translation but also on post-translational modifications, functional proteomic profiling may theoretically yield more direct answers to functional and pharmacological questions than transcriptional profiling alone. However, practical, high-throughput approaches to the study of the functional proteome have not been available until recently. RPPA is a useful tool to identify and validate protein and phospho-proteins [19-23]. Our data suggest that RPPA has the potential to advance our understanding of breast cancer biology and to aid in the identification and validation of useful biomarkers. Our findings validate the importance of ER, PR and HER2. However, seven additional markers including other tyrosine kinase receptors and proliferation markers involved in therapy resistance (EGFR, CCNB1, CCNE1) are part of the 10-protein panel. The combination of 10 markers and the power of the 10 markers as compared to ER/PR and HER2 is novel. The ER, PR and HER2 and the proliferation markers correspond to other breast cancer classifiers such us the intrinsic subtypes or the Oncotype DX Recurrence Score which have also shown that ER, HER2 and proliferation are the most important classifiers, prognostic and predictive markers in breast cancer [1,31]. This demonstrates that RPPA can capture prognostic and predictive differences associated with breast cancer subtypes.

Several factors are important in selection and validation of biomarkers: The analysis platform must be sufficiently robust to detect subtle changes between tumors. Sample sets must be robust enough to reduce pre-analytical data biases and must reflect the intended use of the marker or marker set. Independent sample sets must be used to validate the prognostic and predictive power of biomarkers particularly when many biomarkers are assessed on small sample sets. Lastly, bioinformatics support is essential at all steps in any project. The current study has satisfied all of the requirements mentioned above. RPPA is a robust platform able to detect minimal changes in protein levels [15]. Three large independent sample sets with adequate clinical and outcome information were used for training and testing. Bioinformaticians were closely involved in study design as well as data analyses.

Our findings also have limitations. Patient cohorts received diverse types of systemic treatments and limiting the ability to dissect effects on prognosis from variables that predict endocrine and/or chemotherapy sensitivity. When looking at pCR predictors, all prognostic signatures can reasonably predict pCR, however patients predicted to obtain pCR may have significantly worse survival than those predicted not to respond due to different prognostic variables i.e. HR positivity. So, if our signature is primarily prognostic, its potential utility for selecting chemotherapy sensitivity would be limited; for this reason, validation studies in independent cohorts are needed.

The issues of tumor heterogeneity and the utility of laser captured microdissection were considered in our previous work focusing on the technical assessment of the utility of RPPA for the study of the functional proteome in non-microdissected human breast cancers [20]. This approach used captures information contained in the tumor cells, the stroma and in particular the tumor stroma interaction. The approach of using the complete tumor including interactions of tumor and stroma to classify patients and predict outcomes is the basis for the current transcriptional profiling approaches such as Oncotype Dx or Mammaprint. We have attempted to develop and implement RPPA approaches on microdissected tumors. However, due to a number of technical challenges, this approach is not as robust as study of complete tumors which captures information from the tumor and the stroma as well as tumor stroma interactions.

In summary, we have developed a 10-protein biomarker panel that may have potential utility in the management of patients with breast cancer. Today, it is clear that we should view breast cancer as several distinct diseases. Thus, further work is needed to identify predictors of response to individual therapies that target different clinical and molecular subgroups of breast cancer.

## Abbreviations Page

AIC: Akaike Information Criterion; BCA: Bicinchoninic acid assay; ER: estrogen receptor; FISH: Flourescent in-situ hybridization; FNA: Fine needle aspirate; HR: hormone receptor; IHC: Immunohistochemistry; MDACC: MD Anderson Cancer Center; NST: Neoadjuvant systemic therapy; pCR: Pathologic complete response; PR: Progesterone receptor; PS: Prognostic Score; RFS: Recurrence-free survival; ROC: Receiving operating curve; RPPA: Reverse phase protein array

## Competing interests

Authors declare that they have no competing interests.

## Authors' contributions

AMG-A**: **Contributed samples, Performed all experiments, Analyzed the data, Wrote the manuscript, Funded the experiments. BTH**: **Contributed samples, Performed all experiments, Analyzed the data, Wrote the manuscript, Funded the experiments. FMB**: **Contributed samples, Analyzed the data, Wrote the manuscript. AS**: **Contributed samples, Approved final manuscript. WL**: **Analyzed the data, Approved final manuscript. ZJ**: **Analyzed the data, Approved final manuscript. MSC**: **Contributed samples, Performed experiments. SM**: **Contributed samples, Approved final manuscript. CS**: **Contributed samples, Approved final manuscript. LD**: **Contributed antibodies, Approved final manuscript. RB**: **Contributed antibodies, Approved final manuscript. AL**: **Contributed samples, Approved final manuscript SA**: **Contributed samples, Approved final manuscript. PB**: **Contributed samples, Approved final manuscript. LP**: **Contributed samples, Approved final manuscript. WFS**: **Contributed samples, Approved final manuscript. JA**: **Contributed samples, Approved final manuscript. JO**: **Contributed samples, Approved final manuscript. A-LB-D**: **Contributed samples, Approved final manuscript. GNH**: **Contributed data, Approved final manuscript. KRC**: **Analyzed the data, Approved final manuscript. GBM**: **Contributed samples, Analyzed the data, Wrote the manuscript, Funded the experiments, Approved final manuscript. All authors read and approved the final manuscript.

## Supplementary Material

Additional file 1 **Supplemental Data**. Table S1. Monospecific antibodies used in this study, Figure S1. Unsupervised clustering of 712 breast cancers (Training Set) using quantification data for 146 proteins derived using reverse phase protein arrays. Figure S2. Kaplan Meier Survival Curves for RFS of the 541 patients according to their subgroup classification. Table S2. Distribution of tumors by breast cancer subtype and prognostic group (PG) according to the 10 marker signature Figure S3.A plot of the deviance residuals from the cox PH model (using the prognostic score and grade as the predictors to model relapse free survival in the training data set) against the prognostic score. Figure S4. A plot of the deviance residuals from the logistic model (using the prognostic score and grade to predict the probability of pCR in the FNA test data set) against the predicted probability of pCR. Click here for file

Additional file 2 **Expansion of the Statistical methods**. More detailed description of the statistical methods with the corresponding references. Click here for file

Additional file 3 **Breast cancer classifier via a logistic-regression decision tree**. Locked logistic-regression tree used for validation Click here for file
