# Supplementary material for: Functional proteomics can define prognosis and predict pathologic complete response in patients with breast cancer
Source: Clin Proteomics. 2011 Jul 8;8(1):11. doi: 10.1186/1559-0275-8-11 (PMC3170272; doi:10.1186/1559-0275-8-11)
Supplement: Additional file 1 — Supplemental Data. Table S1. Monospecific antibodies used in this study, Figure S1. Unsupervised clustering of 712 breast cancers (Training Set) using quantification data for 146 proteins derived using reverse phase protein arrays. Figure S2. Kaplan Meier Survival Curves for RFS of the 541 patients according to their subgroup classification. Table S2. Distribution of tumors by breast cancer subtype and prognostic group (PG) according to the 10 marker signature Figure S3.A plot of the deviance residuals from the cox PH model (using the prognostic score and grade as the predictors to model relapse free survival in the training data set) against the prognostic score. Figure S4. A plot of the deviance residuals from the logistic model (using the prognostic score and grade to predict the probability of pCR in the FNA test data set) against the predicted probability of pCR. [file 1559-0275-8-11-S1.DOC]

**Supplemental Table 1. Monospecific antibodies used in this study.**

| **Antibody name** | **Protein name** | **Company** | **Catalog number** | **Host** | **Dilution** |
| --- | --- | --- | --- | --- | --- |
| 14-3-3 Beta | 14-3-3 Beta | Santa Cruz Biotechnology, Inc. | SC-628 | Rabbit | 1 in 5000 |
| 14-3-3 Zeta | 14-3-3 Zeta | Santa Cruz Biotechnology, Inc. | SC-1019 | Rabbit | 1 in 1200 |
| 4EBP1 | 4E Binding Protein 1 | Cell Signaling Technology, Inc. | CS 9452 | Rabbit | 1 in 100 |
| 4EBP1pT37/T46 | 4EBP1 phosphorylation at T37/T46 | Cell Signaling Technology, Inc. | CS 9459 | Rabbit | 1 in 100 |
| 4EBP1pS65 | 4EBP1 phosphorylation at S65 | Cell Signaling Technology, Inc. | CS 9451 | Rabbit | 1 in 200 |
| 4EBP1pS65 (mono) | 4EBP1 phosphorylation at S65 | Cell Signaling Technology, Inc. | CS 9456 | Rabbit | 1 in 250 |
| 4EBP1pT70 | 4EBP1 phosphorylation at T70 | Cell Signaling Technology, Inc. | CS 9455 | Rabbit | 1 in 150 |
| AcCoA | Acetyl CoA Carboxylase | Epitomics, Inc. | 1768-1 | Rabbit | 1 in 250 |
| AcCoApS79 | AcCoA phosphorylation at S79 | Cell Signaling Technology, Inc. | CS 3661 | Rabbit | 1 in 250 |
| AIB1 | Amplified in Breast Cancer 1 | BD Biosciences | 611105 | Mouse | 1 in 50 |
| Akt | Protein Kinase B | Cell Signaling Technology, Inc. | CS 9272 | Rabbit | 1 in 250 |
| AktpS308 | Akt phosphorylation at S308 | Cell Signaling Technology, Inc. | CS 9275 | Rabbit | 1 in 250 |
| AktpS473 | Akt phosphorylation at S473 | Cell Signaling Technology, Inc. | CS 9271 | Rabbit | 1 in 250 |
| alpha.actin | Alpha actin | Epitomics, Inc. | 1184-1 | Rabbit | 1 in 200 |
| AMPK | AMPK | Cell Signaling Technology, Inc. | CS 2532 | Rabbit | 1 in 250 |
| AMPKpS172 | AMPK phosphorylation at S172 | Cell Signaling Technology, Inc. | CS 2535 | Rabbit | 1 in 250 |
| AR | Androgen Receptor | Epitomics, Inc. | 1852-1 | Rabbit | 1 in 200 |
| β catenin | B catenin | Cell Signaling Technology, Inc. | CS 9562 | Rabbit | 1 in 300 |
| BAD | BAD | Cell Signaling Technology, Inc. | CS 9292 | Rabbit | 1 in 200 |
| BADpS112 | BAD phosphorylation at S112 | Cell Signaling Technology, Inc. | CS 9296 | Mouse | 1 in 200 |
| BCL11A | BCL11A | SDI | 2172 | Rabbit | 1 in 1500 |
| bcl2 | bcl2 | Dako | M0887 | Mouse | 1 in 200 |
| BCLpS70 | bcl2 phosphorylation at S70 | Cell Signaling Technology, Inc. | CS 2827 | Rabbit | 1 in 200 |
| BIM | BIM | Epitomics, Inc. | 1036 | Rabbit | 1 in 250 |
| BRCA1 | BRCA1 | Upstate Biotechnology, Inc. | 07-434 | Rabbit | 1 in 1000 |
| BRCA2 | BRCA2 | Cell Signaling Technology, Inc. | CS 9012 | Rabbit | 1 in 300 |
| caveolin 1 | Caveolin 1 | Cell Signaling Technology, Inc. | CS 3232 | Rabbit | 1 in 250 |
| CCNB1 | Cyclin B1 | Epitomics, Inc. | 1495-1 | Rabbit | 1 in 500 |
| CCND1 | Cyclin D1 | Santa Cruz Biotechnology, Inc. | SC-718 | Rabbit | 1 in 1000 |
| CCNE1 | Cyclin E1 | Santa Cruz Biotechnology, Inc. | SC-247 | Mouse | 1 in 500 |
| CCNE2 | Cyclin E2 | Epitomics, Inc. | 1142-1 | Rabbit | 1 in 250 |
| CD20 | CD20 | Epitomics, Inc. | 1632 | Rabbit | 1 in 125 |
| CD31 | CD31 | Dako | M0823 | Mouse | 1 in 500 |
| CD4 | CD4 | R&D Systems | MAB3791 | Mouse | 1 in 1500 |
| cdk2 | Cyclin dependent kinase 2 | BD Biosciences | 610145 | Mouse | 1 in 50 |
| CDK4 | Cyclin dependent kinase 4 | Cell Signaling Technology, Inc. | CS 2906 | Rabbit | 1 in 250 |
| cjun | Cjun | Cell Signaling Technology, Inc. | CS 9165 | Rabbit | 1 in 250 |
| cJUNpS73 | cjun phosphorylation at S73 | Cell Signaling Technology, Inc. | CS 9164 | Rabbit | 1 in 150 |
| ckit | Ckit | Cell Signaling Technology, Inc. | CS 3392 | Rabbit | 1 in 150 |
| cleaved caspase 7 | Cleaved caspase 7 (Asp198) | Cell Signaling Technology, Inc. | CS 9491 | Rabbit | 1 in 150 |
| cleaved PARP | Cleaved PARP (Asp214) | Cell Signaling Technology, Inc. | CS 9546 | Mouse | 1 in 250 |
| cmyc | Cmyc | Cell Signaling Technology, Inc. | CS 9402 | Rabbit | 1 in 150 |
| Collagen V | Collagen V | Santa Cruz Biotechnology, Inc. | SC-20648 | Rabbit | 1 in 1000 |
| Collagen VI | Collagen VI | Santa Cruz Biotechnology, Inc. | SC-20649 | Rabbit | 1 in 750 |
| COX2 | COX2 | Epitomics, Inc. | 2169-1 | Rabbit | 1 in 500 |
| COMT | Catechol-O-methyltransferase | SDI | 1671 | Rabbit | 1 in 15000 |
| Connexin | Connexin | SDI | 1716 | Rabbit | 1 in 1500 |
| CXXC6 | CXXC6 | SDI | 2126 | Rabbit | 1 in 1750 |
| E cadherin | E cadherin | Cell Signaling Technology, Inc. | CS 4065 | Rabbit | 1 in 200 |
| EGFR | Epidermal growth factor receptor | Santa Cruz Biotechnology, Inc. | SC-03 | Rabbit | 1 in 200 |
| EGFRpY1045 | EGFR phosphorylation at Y1045 | Cell Signaling Technology, Inc. | CS 2237 | Rabbit | 1 in 100 |
| EGFRpY922 | EGFR phosphorylation at Y992 | Cell Signaling Technology, Inc. | CS 2235 | Rabbit | 1 in 100 |
| EIG121 | EIG121 / Maba1 | Russell Broaddus at MDACC | N/A | Rabbit | 1 in 200 |
| EN1 | Engrailed-1 | SDI | 2104 | Rabbit | 1 in 1000 |
| ER | Estrogen receptor alpha | Lab Vision Coorporation (formerly Neomarkers) | Sp1 | Rabbit | 1 in 250 |
| ERK2 | Mitogen-activated protein kinase | Cell Signaling Technology, Inc. | SC-154 | Rabbit | 1 in 250 |
| ERpS118 | ER phosphorylation at S118 | Epitomics, Inc. | 1091-1 | Rabbit | 1 in 200 |
| ERpS167 | ER phosphorylation at S167 | Epitomics, Inc. | 2492-1 | Rabbit | 1 in 200 |
| ETV6 | ETV6 | SDI | 2101 | Rabbit | 1 in 400 |
| FANCA | Fanconi anemia, complementation group A | SDI | 2127 | Rabbit | 1 in 2000 |
| FANCE | Fanconi anemia, complementation group E | SDI | 2131 | Rabbit | 1 in 5000 |
| FGFR1 | Fibroblast Growth Factor Receptor 1 | Santa Cruz Biotechnology, Inc. | SC-7945 | Rabbit | 1 in 250 |
| FGFR2 | Fibroblast Growth Factor Receptor 2 | SDI | 2182 | Rabbit | 1 in 600 |
| Fibronectin | Fibronectin | Epitomics, Inc. | 1574 | Rabbit | 1 in 5000 |
| FKHRL1pS318/S321 | FKHRL1 phosphorylation at S318/S321 | Cell Signaling Technology, Inc. | CS 9465 | Rabbit | 1 in 1000 |
| Fortilin | Fortilin | Ken Fujise at UT Houston | N/A | Rabbit | 1 in 3000 |
| GABApi | Gaba aminobutyric acid pi | SDI | 2103 | Rabbit | 1 in 200 |
| GATA3 | GATA3 | BD Biosciences | 558686 | Mouse | 1 in 200 |
| Gelsolin | Gelsolin | SDI | 1718 | Rabbit | 1 in 1000 |
| GSK3 | Glycogen synthase kinase 3 beta | Santa Cruz Biotechnology, Inc. | SC-7291 | Mouse | 1 in 1000 |
| GSK3pS21/S99 | GSK3 phosphorylation at S21/S9 | Cell Signaling Technology, Inc. | CS 9331 | Rabbit | 1 in 250 |
| HDAC7 | Histone deacetylase 7 | Abcam, Inc. | 53101 | Rabbit | 1 in 400 |
| HER2 | Human epidermal receptor 2 | Epitomics, Inc. | 1148-1 | Rabbit | 1 in 250 |
| HER2pY1248 | HER2 phosphorylation at Y1248 | Upstate Biotechnology, Inc. | 06-229 | Rabbit | 1 in 750 |
| IGFBP2 | IGF1 binding protein 2 | Cell Signaling Technology, Inc. | CS3922 | Rabbit | 1 in 150 |
| IGF1R | Insulin-like growth factor receptor 1 | Cell Signaling Technology, Inc. | CS 3027 | Rabbit | 1 in 500 |
| IGFRp Y1135/Y1136 | IGF1R phosphorylation at Y1135/Y1136 | Cell Signaling Technology, Inc. | CS 3024 | Rabbit | 1 in 200 |
| JAZf1 | JAZf1 | SDI | 2138 | Rabbit | 1 in 1000 |
| JNK | cjun N terminal Kinase | Santa Cruz Biotechnology, Inc. | SC-474 | Rabbit | 1 in 200 |
| JNKp T183/Y185 | JNK phosphorylation at T183/Y185 | Cell Signaling Technology, Inc. | CS 9251 | Rabbit | 1 in 150 |
| KIT | KIT | SDI | 2036 | Rabbit | 1 in 15000 |
| LKB1 | LKB1 | Abcam, Inc. | 15095 | Rabbit | 1 in 200 |
| LKB1pS428 | LKB1 phosphorylation at S428 | Cell Signaling Technology, Inc. | CS 3051 | Mouse | 1 in 200 |
| MALT1 | MALT1 | SDI | 2155 | Rabbit | 1 in 1500 |
| MAML2 | MAML2 | SDI | 2136 | Mouse | 1 in 1500 |
| MAPKpS428 | MAPK1/2 phosphorylation at S428 | Cell Signaling Technology, Inc. | CS 4377 | Rabbit | 1 in 1000 |
| MEK1 | MAPK/ERK kinase 1 | Epitomics, Inc. | 1235-1 | Rabbit | 1 in 15000 |
| MEK12pT217/T221 | MEK1/2 phosphorylation at T217/T221 | Cell Signaling Technology, Inc. | CS 9121 | Rabbit | 1 in 800 |
| MGMT | Methylguanine Methyltransferase | Chemicon | 16200 | Mouse | 1 in 100 |
| MLLT10 | MLLT10 | SDI | 2116 | Rabbit | 1 in 400 |
| mTOR | Mammalian target of rapamycin | Cell Signaling Technology, Inc. | CS 2983 | Rabbit | 1 in 400 |
| MYH11 | MYH11 | SDI | 2137 | Rabbit | 1 in 2000 |
| Ncadherin | Ncadherin | Cell Signaling Technology, Inc. | CS 4061 | Rabbit | 1 in 100 |
| NCKIPSI | NCKIPSI | SDI | 2117 | Rabbit | 1 in 1000 |
| NOTCH3 | NOTCH3 | Santa Cruz Biotechnology, Inc. | SC-5593 | Rabbit | 1 in 100 |
| p110alpha | p110alpha subunit of phosphatidylinositol-3-kinase | Epitomics, Inc. | 1683-1 | Rabbit | 1 in 500 |
| p21 | p21 | Santa Cruz Biotechnology, Inc. | SC-397 | Rabbit | 1 in 250 |
| p27 | p27 | Santa Cruz Biotechnology, Inc. | SC-527 | Rabbit | 1 in 500 |
| p38 | p38 MAPK | Cell Signaling Technology, Inc. | CS 9212 | Rabbit | 1 in 300 |
| p38pT180/T182 | p38 MAPK phosphorylation at T180/T182 | Cell Signaling Technology, Inc. | CS 9211 | Rabbit | 1 in 250 |
| p53 | p53 | Cell Signaling Technology, Inc. | CS 9282 | Rabbit | 1 in 3000 |
| p53pS15 | p53 phosphorylation at S15 | Cell Signaling Technology, Inc. | CS 9284 | Rabbit | 1 in 5000 |
| p70S6 Kinase | p70S6 Kinase | Epitomics, Inc. | 1494-1 | Rabbit | 1 in 500 |
| p70S6KpT389 | p70S6 Kinase phosphorylation at T389 | Cell Signaling Technology, Inc. | CS 9205 | Rabbit | 1 in 200 |
| p90RSKpS380 | p90RSK phosphorylation at S380 | Cell Signaling Technology, Inc. | CS 9341 | Rabbit | 1 in 400 |
| PAI1 | Plasminogen activator inhibitor-1 | BD Biosciences | 612024 | Mouse | 1 in 1000 |
| pcmycT58/S62 | cmyc phosphorylation at T58/S62 | Cell Signaling Technology, Inc. | CS 9401 | Rabbit | 1 in 150 |
| PCNA | Proliferating Cell Nuclear Antigen | Abcam, Inc. | 29 | Mouse | 1 in 2000 |
| PDK1 | Phosphoinositide Dependent Kinase 1 | Cell Signaling Technology, Inc. | CS 3062 | Rabbit | 1 in 250 |
| PDK1pS241 | PDK1 phosphorylation at S241 | Cell Signaling Technology, Inc. | CS 3061 | Rabbit | 1 in 500 |
| PKCalpha | Protein Kinase C alpha | Upstate Biotechnology, Inc. | 05-154 | Mouse | 1 in 2000 |
| PKCaphapS657 | PKCalpha phosphorylation at S657 | Upstate Biotechnology, Inc. | 06-822 | Rabbit | 1 in 3000 |
| PML | PML | SDI | 2114 | Rabbit | 1 in 3000 |
| pmTORS2448 | mTOR phosphorylation at S2448 | Cell Signaling Technology, Inc. | CS 2971 | Rabbit | 1 in 150 |
| PR | Progesterone receptor | Epitomics, Inc. | 1483-1 | Rabbit | 1 in 400 |
| PSAT1 | PSAT1 | SDI | 2102 | Rabbit | 1 in 2000 |
| PTCH | Patche | SDI | 2113 | Rabbit | 1 in 800 |
| PTEN | PTEN | Cell Signaling Technology, Inc. | CS 9552 | Rabbit | 1 in 500 |
| Rab25 | Rab25 | Courtesy Dr. Kwai Wa Cheng, MDACC | Covance | Rabbit | 1 in 4000 |
| Rb | Retinoblastoma | Cell Signaling Technology, Inc. | CS 9309 | Mouse | 1 in 3000 |
| RBM15 | RBM15 | SDI | 2139 | Rabbit | 1 in 2500 |
| RbpS807/S811 | Rb phosphorylation at S807/S811 | Cell Signaling Technology, Inc. | CS 9308 | Rabbit | 1 in 250 |
| ROPN1 | Rhophilin 1 | SDI | 2105 | Rabbit | 1 in 600 |
| S6 | S6 ribosomal protein | Cell Signaling Technology, Inc. | CS 2217 | Rabbit | 1 in 200 |
| S6pS235/S236 | S6 phosphorylation at S235/S236 | Cell Signaling Technology, Inc. | CS 2211 | Rabbit | 1 in 3000 |
| S6pS240/S244 | S6 phosphorylation at S240/S244 | Cell Signaling Technology, Inc. | CS 2215 | Rabbit | 1 in 3000 |
| SGK | Serum Glucocorticoid Kinase | Cell Signaling Technology, Inc. | CS 3272 | Rabbit | 1 in 250 |
| SGKpS78 | SGK phosphorylation at S78 | Cell Signaling Technology, Inc. | CS 3271 | Rabbit | 1 in 250 |
| src | Src | Upstate Biotechnology, Inc. | 05-184 | Mouse | 1 in 200 |
| srcpY416 | src phosphorylation at Y416 | Cell Signaling Technology, Inc. | CS 2101 | Rabbit | 1 in 150 |
| srcpY527 | src phosphorylation at Y527 | Cell Signaling Technology, Inc. | CS 2105 | Rabbit | 1 in 400 |
| stat3 | Signal transducer and activator of transcription 3 | Upstate Biotechnology, Inc. | 06-596 | Rabbit | 1 in 500 |
| stat3pS705 | stat3 phosphorylation at S705 | Cell Signaling Technology, Inc. | CS 9131 | Rabbit | 1 in 500 |
| stat3pS727 | stat3 phosphorylation at S727 | Cell Signaling Technology, Inc. | CS 9134 | Rabbit | 1 in 250 |
| stat6pY641 | stat6 phosphorylation at Y641 | Cell Signaling Technology, Inc. | CS 9361 | Rabbit | 1 in 150 |
| stathmin | Stathmin | Epitomics, Inc. | 1972-1 | Rabbit | 1 in 500 |
| Tau | Microtubule Associated Protein Tau | Santa Cruz Biotechnology, Inc. | SC-58855 | Mouse | 1 in 150 |
| Tau | Microtubule Associated Protein Tau | Upstate Biotechnology, Inc. | 05-348 | Mouse | 1 in 150 |
| TAZ | TAZ | Abcam, Inc. | 3961 | Rabbit | 1 in 250 |
| TAZpS89 | TAZ phosphorylation at S89 | Santa Cruz Biotechnology, Inc. | SC-17610R | Rabbit | 1 in 250 |
| Telomerase | Telomerase | SDI | 1706 | Rabbit | 1 in 250 |
| TopoII | Topoisomerase II | Abcam, Inc. | 45175 | Rabbit | 1 in 100 |
| TSC2 | Tuberous Sclerosis Kinase 2 | Epitomics, Inc. | 1613-1 | Rabbit | 1 in 500 |
| TSC2pT1462 | TSC2 phosphorylation at T1462 | Cell Signaling Technology, Inc. | CS 3617 | Rabbit | 1 in 200 |
| VEGFR2 | KDR2 / VEGF Receptor 2 | Cell Signaling Technology, Inc. | CS 2479 | Rabbit | 1 in 700 |
| XIAP | X linked inhibitor of apoptosis | Cell Signaling Technology, Inc. | CS 2042 | Rabbit | 1 in 200 |
| YAP | YAP | Santa Cruz Biotechnology, Inc. | 15407 | Rabbit | 1 in 500 |
| YB1 | Y-Box Binding Protein 1 | SDI | 1725 | Rabbit | 1 in 2500 |
| YKL40 | YKL40 | Ken Aldape at MDACC |  | Rabbit | 1 in 3000 |

Companies: Abcam, Inc. (Cambridge, MA), BD Biosciences (San Jose, CA), Cell Signaling Technology, Inc. (Danvers, MA), Chemicon International Inc. (Billerica, MA), Dako (Carpinteria, CA), Epitomics, Inc. (Burlingame, CA), R&D Systems, (Minneapolis, MN), Santa Cruz Biotechnology, Inc. (Santa Cruz, CA), SDI (Newark, DE), Upstate Biotechnology (Millipore) Inc. (Billerica, MA).

**Supplemental Figure 1. Unsupervised clustering of 712 breast cancers (Training Set) using quantification data for 146 proteins derived using reverse phase protein arrays.** The 146 protein endpoints used in the analysis stratified the breast cancer samples into six major groups. In addition to the expected hormone receptor (HR)-positive, and HER2-positive groups, two groups were characterized by overexpression of proteins including cyclins B1 and E1 as well as components of the protein synthesis machinery including phosphorylated S6 ribosomal protein and 4EBP1. These groups were a hormone receptor-negative and HER2-negative (i.e. triple receptor-) negative, and a HR-positive group. Other groups were composed of tumors with overexpression of a group of stromal markers including collagen VI, CD31 and caveolin 1, or defined by an up-regulation of a relatively large number of proteins and phospho-proteins that span several mechanistic pathways.

**HR-Positive**

**Translation activity**

**HR-Positive**

**Supplemental Figure 2.** Kaplan Meier Survival Curves for RFS of the 541 patients according to their subgroup classification.

**Supplemental Table 2.** Distribution of tumors by breast cancer subtype and prognostic group (PG) according to the 10 marker signature.

|  | **HR positive and HER2negative** | **HER2 positive** | **Triple receptor negative** | **Unknown** |
| --- | --- | --- | --- | --- |
| **PG1** | **144** | **4** | **5** | **0** |
| **PG2** | **127** | **11** | **3** | **0** |
| **PG3** | **46** | **3** | **5** | **1** |
| **PG4** | **10** | **6** | **88** | **1** |
| **PG5** | **53** | **18** | **82** | **1** |
| **PG6** | **3** | **100** | **1** | **0** |

**Supplemental Figure 3.** A plot of the deviance residuals from the cox PH model (using the prognostic score and grade as the predictors to model relapse free survival in the training data set) against the prognostic score. There are no extreme outliers and the residuals are basically symetric and random from left to right. This corresponding to model ‘CoxTest’.

**
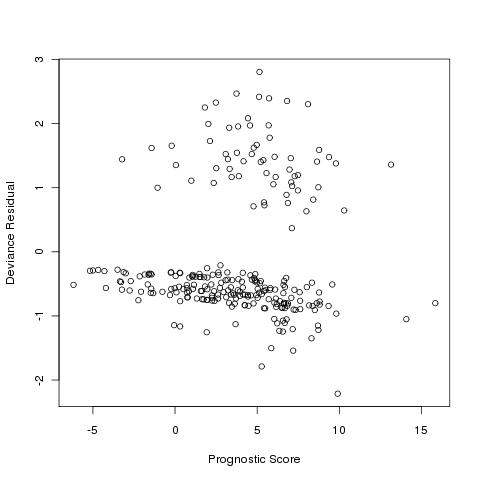
**

**Supplemental Figure 4.** A plot of the deviance residuals from the logistic model (using the prognostic score and grade to predict the probability of pCR in the FNA test data set) against the predicted probability of pCR. We do not see extreme outliers. This corresponds to model “LR-FNA”.

**
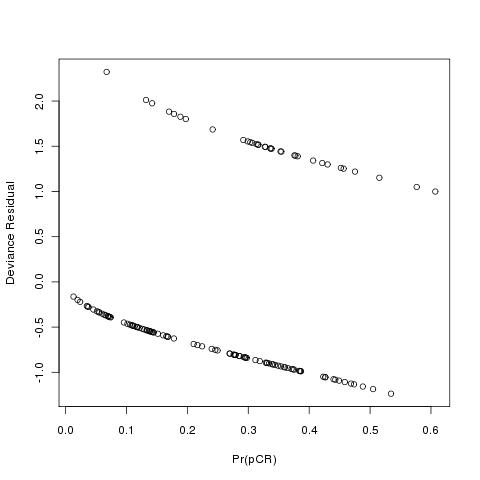
**

**F**
