# Supplementary material for: Functional proteomics can define prognosis and predict pathologic complete response in patients with breast cancer
Source: Clin Proteomics. 2011 Jul 8;8(1):11. doi: 10.1186/1559-0275-8-11 (PMC3170272; doi:10.1186/1559-0275-8-11)
Supplement: Additional file 2 — Expansion of the Statistical methods. More detailed description of the statistical methods with the corresponding references. [file 1559-0275-8-11-S2.DOCX]

**Expansion of Statistical Methods**

**Identification of Prognostic Groups**

Unsupervised clustering analysis, using the uncentered correlation distance metric (1) and Ward’s linkage rule (2), was applied to the training set to define groups and correlate them with breast cancer subtypes defined by cell surface markers and by transcriptional profiling. To develop a more limited set of markers for breast cancer classification and outcomes prediction, we used a hypothesis-driven approach by selecting markers according to their functional assignments and subsequently performed a supervised proteomic clustering analysis to optimize the selection of groups with the most distinct recurrence-free survival (RFS) outcomes. Wee hypothesized that three functions would most impact the behavior and therapy responsiveness in breast cancer: ER function, grade/proliferation, and receptor tyrosine kinase activity. From the initial146 antibodies, we then selected all markers within these three functional categories and tested multiple combinations of these markers requiring that a minimum of one marker per functional category remain in each model. Selected proteins groups were as follows: ER function (ER, ERpS118, ERpS167, PR, AR, EIG121, Bcl2, GATA3, IGF1R, and IGFBP2), grade/proliferation (CCNB1, CCND1, CCNE1, CCNE2, and PCNA), and receptor tyrosine kinase activity (cKit, EGFR, EGFRp1045, EGFRp922, HER2, HER2p1248, FGFR1, FGFR2, IGF1R, IGFRpY1135/Y1136). We then visualized the RFS curves to select the marker set or model that was associated with the clearest differences in RFS between the identified groups in the training set. Because of multiple testing and the possibility of false discovery, this model was then applied to a test set.

RFS was estimated according to the Kaplan-Meier method and compared between groups using the log-rank statistic. Cox proportional Hazard Models were fitted using the proteomic subgroups, the selected markers and the clinical variables.

**Decision trees**

We constructed a model to predict the classes discovered by hierarchical clustering using a binary decision tree with a logistic regression model at each node. The split at each node was a union of some of the classes. Protein-by-protein two-sample t-tests between the two halves of the split were computed. The proteins were ordered by p-value and then were added one at a time into a logistic regression model until a desired prediction accuracy was achieved. In order to avoid overfitting data, a default precision accuracy of 95% was set at each node. Finally, the Akaike Information Criterion (AIC) was used to eliminate redundant terms from the logistic regression model (3). The final model accurately placed 89% of the samples into the correct one of the six categories.

**Validation of Prognostic Groups for RFS**

The coefficients of the model, which used logistic regression at each node of a decision tree to place samples in one of six classes (or prognostic groups) were finalized and locked on 2 September 2008. The model was applied to a blinded set of RPPA data from the test set of 168 samples on 18 November 2008. An implementation of the model in R was provided to an independent analyst, along with the class predictions. The independent analyst was provided with the unblinded clinical data after implementation of the model on 18 November 2008. Cox proportional hazards models were then constructed using the predicted classes as covariates to test their association with RFS.

**Agreement between clusters and predictions**

We then applied the algorithm to the last sample set (132 FNA from MDACC) and correlated the groups with response to NST. We clustered the FNA samples as above and compared these clusters to the class labels predicted by the decision tree model with Cohen’s kappa statistic (4,5). In order to apply Cohen’s kappa statistic, it was necessary to reorder the rows (predicted categories) and columns (observed clusters) in a contingency table. The unsupervised clustering methods that we used produce binary trees, which can freely be reordered (without changing the underlying mathematical structure) by exchanging the left and right branches at any point in the tree. For example, the relative positions of the red (PG1) and orange (PG2) clusters in the training set (Figure 1A) are reversed in the test set (Figure 1B). In order to uniquely identify the predicted categories among the new clusters, we chose the ordering that maximized the diagonal terms in the contingency matrix; this corresponds to a maximum-likelihood estimate for matching categories. We note that the chi-squared statistic is not sensitive to this reordering of rows and columns, although Cohen’s kappa is affected. We used Cohens’ kappa statistic for five different comparisons.

1. All proteins vs. Hypothesis-driven proteins (training set)

We clustered the training samples twice, first using all 146 proteins and then using only the ten proteins selected for our hypothesis-driven approach. The contingency table is as follows:

|  | A1 | A3 | A4 | A2 | A5 | A6 |
| --- | --- | --- | --- | --- | --- | --- |
| PG1 | 58 | 45 | 22 | 0 | 28 | 0 |
| PG2 | 29 | 81 | 10 | 0 | 17 | 4 |
| PG3 | 7 | 1 | 29 | 1 | 17 | 0 |
| PG4 | 1 | 0 | 3 | 91 | 10 | 0 |
| PG5 | 3 | 2 | 66 | 29 | 54 | 0 |
| PG6 | 4 | 7 | 1 | 12 | 11 | 69 |

Here the “PG” categories are as in the main manuscript, and arise from clustering using the ten proteins. The “A” categories are the clusters using all proteins. The agreement is highly significant (kappa = 0.444, p < 1e-16). However, since a relatively large number of samples still changed categories, we chose to proceed using the clusters defined by the ten proteins.

2. Clusters vs. Decision-tree model (Training set)

We checked the agreement between the actual clusters (Figure 1A) and the predictions from the decision tree model on the training set that was used to derive the model. Not surprisingly, the agreement was extremely good (kappa = 0.861, p < 1e-16), with 87% of the samples being placed into the correct cluster by the decision tree model.

3. Clusters vs. Decision-tree predictions (Test set)

We clustered the 168 samples in the test data set (Figure 1B), and we also predicted classes using the decision-tree model that was learned from the training set. The contingency table is as follows.

|  | Cluster4 | Cluster2 | Cluster5 | Cluster1 | Cluster3 | Cluster6 |
| --- | --- | --- | --- | --- | --- | --- |
| PG1 | 26 | 5 | 0 | 2 | 0 | 0 |
| PG2 | 1 | 42 | 0 | 0 | 0 | 2 |
| PG3 | 4 | 2 | 9 | 0 | 0 | 0 |
| PG4 | 0 | 1 | 0 | 22 | 1 | 0 |
| PG5 | 0 | 0 | 12 | 4 | 4 | 0 |
| PG6 | 0 | 0 | 0 | 0 | 19 | 12 |

Here the “PG” categories are the one predicted by the model, and the “Cluster” categories are the actual clusters on the test data. The agreement is again extremely significant (kappa = 0.614; P < 1e-16).

4. Clusters vs. Decision-tree predictions (256 FNA samples)

We clustered the 256 FNA samples (Figure 1B) and also predicted the classes using the decision tree model. The contingency table is as follows

|  | Cluster5 | Cluster1 | Cluster6 | Cluster2 | Cluster3 | Cluster4 |
| --- | --- | --- | --- | --- | --- | --- |
| PG1 | 14 | 5 | 0 | 0 | 0 | 0 |
| PG2 | 0 | 42 | 0 | 0 | 0 | 1 |
| PG3 | 6 | 15 | 38 | 0 | 13 | 0 |
| PG4 | 0 | 0 | 1 | 40 | 0 | 1 |
| PG5 | 0 | 0 | 5 | 5 | 36 | 0 |
| PG6 | 0 | 0 | 2 | 0 | 9 | 23 |

Again, the “PG” categories are predicted by the model, and the “Clusters” are observed. The agreement is excellent (kappa = 0.700, p < 1e-16).

5 Clusters vs. Decision-tree predictions (132 FNA samples)

We repeated the previous analysis on the subset of 132 FNA samples from patients who had all received the same treatment. The results wer again excellent (kappa = 0.664, p < 1e-16).

**Validation of Prognostic Groups for pCR**

As noted, we applied the algorithm to the last sample set (132 FNA from MDACC) and correlated the groups with response to NST. We clustered the FNA samples as above and compared these clusters to the class labels predicted by the decision tree model with Cohen’s kappa statistic (4,5). Using the predicted prognostic groups, we developed a Bayesian model to estimate the posterior probability of pCR in each patient group. We modeled the pCR rates as coming from a beta-binomial distribution by assuming that the response in the sample group $PG_{j}$is binomial with pCR rate given by $\theta_{j}$, where $\theta_{j}$comes from a common beta distribution ($\theta_{j}\sim Beta(\alpha,\beta)$). We put an uninformative prior on the unknown parameters α and β by transforming to the preferred parameter space that uses the log ratio ($x=\log(\alpha/\beta)$) and the log of the sum ($y=\log(\alpha+\beta)$) (6). Given the actual data (number of responses *k* in a group of *n* patients), we then computed joint posterior probabilities for the parameters. We transformed the parameters from *x-y*-space back to α-β-space to compute the expected value (mean) and equivalent sample size of the beta distribution.

**Development of a Prognostic Score and its Application to Prediction of pCR**

We next converted the six prognostic groups into a continuous prognostic score by fitting an ordinal regression model on the first data set (training set, 712 tumors) (7). The response variable was the prognostic group where levels were ordered by increasing risk of recurrence as shown in the Kaplan-Meier curves in Figure 1D. (Specifically, we used the order PG2 < PG3 < PG1 < PG5 < PG4 < PG6.) The predicting variables were the selected protein markers. The resulting continuous score is a weighted linear combination of the relative concentration of the protein markers (ER: -0.2841, PR: -1.3038, Bcl2: 0.0826, GATA3: -0.6876, CCNB1: 0.5169, CCNE1: 0.1000, EGFR: 0.4321, HER2: 0.5564, HER2p1248: 0.8284, EIG121: 0.2424). The model was tested using the second data set (test set, 168 tumors). The score was associated with the RFS estimates by the Cox proportional hazards model. With satisfactory prediction of RFS in this test set, we applied the model to the NST treated FNA data set (132 patients). The same predictions and tests were performed except that a logistic regression model was fitted using the NST response as the binary response variable (pCR vs. residual disease), instead of the Cox model. The prediction of response was evaluated by a receiver operating characteristics (ROC) curve.

**Models for Recurrence-Free Survival and Likelihood of Pathologic Complete Response**

A Cox proportional hazards model was fit using each of the following covariates: prognostic group, tumor size, histologic grade, node status, each of the 10 protein markers, and the prognostic score to estimate their association with RFS. In these models, the hazard ratio is relative to the lowest level of the covariate if the latter is continuous (prognostic score, size, node and grade), and is the fold increase of hazard with unit increase of the covariate if the latter is continuous (the protein markers and prognostic score). Using the same covariates, a logistic regression model was fit to estimate the association of each covariate with pCR. Stepwise multivariate model selection based on the Akaike Information Criterion (8,9) was used to determine the combination of covariates for the multivariate models. All the statistical analysis in this study was performed in R 2.8.1 (10).

**References**

1. Eisen MB, Spellman PT, Brown PO, Botstein D. Cluster analysis and display of genome-wide expression patterns. *Proc Natl Acad Sci U S A*. 1998;95:14863-14868.
2. Ward JH. Hierachical grouping to optimize an objective function. *J Am Statist Assoc.* 1963;58:236-244.
3. Akaike H. A new look at the statistical model identification. *IEEE Transactions on Automatic Control.* 1974;19: 716–723.
4. Wolfrum C. Appearance of quasiequivalent solutions, in a generalization of the Kruskal scale technic, to metric spaces using a Minkowski metric. *Arch Psychol.* 1976;128:96-111.
5. Landis JR Koch GG. The measurement of observer agreement for categorical data. *Biometrics.* 1977;33:159-174.
6. Gelman A, Carlin JB, Stern HS, Rubin DB. Bayesian Data Analysis, 2nd ed. Chapman & Hall, Boca Raton, 2004. (Section 5.3, pages 125-131).
7. Verweij PJM, Van Houwelingen JC: Penalized likelihood in Cox regression. *Stat in Med*. 1994;13:2427-2436.
8. Hastie TJ, Pregibon D. Generalized linear models. In JM Chambers and TJ Hastie, Statistical Models in S. Wadsworth & Brooks/Cole Computer Science Series (Hardcover). Chapman & Hall/CRC. Boca Raton, Fla. 1991. (Chapter 6, pages 195-248).
9. Venables WN, Ripley BD. Generalized Linear Models. In WN Venables, BD Ripley Modern Applied Statistics with S (4th ed). Springer-Verlag, New York, 2002. (Chapter 7, pages 183-208).
10. R Development Core Team (2008). R: A language and environment for statistical computing R Foundation for Statistical Computing, Vienna, Austria. ISBN 3-900051-07-0, URL http://www.R-project.org.
