# Supplementary material for: Functional proteomics can define prognosis and predict pathologic complete response in patients with breast cancer
Source: Clin Proteomics. 2011 Jul 8;8(1):11. doi: 10.1186/1559-0275-8-11 (PMC3170272; doi:10.1186/1559-0275-8-11)
Supplement: Additional file 3 — Breast cancer classifier via a logistic-regression decision tree. Locked logistic-regression tree used for validation [file 1559-0275-8-11-S3.PDF]

Breast cancer classifier via a logistic-regression decision tree

Kevin R. Coombes

2 September 2008; updated on 22 June 2009

1 Introduction

As part of our analysis of the training set of 712 breast tumors, we used supervised clustering to select a set of 10 markers that clustered samples in a manner that appeared to be related to survival. By performing robust hierarchical clustering using those ten markers, we defined six distinct subtypes of breast cancer. In order to convert the exploratory tools of clustering into a complete predictive model, we arranged those six subtypes into a tree (as reflected by the top branches of the hierarchical dendrogram and shown in Figure 1). We then developed a predictive model that used separate logistic regression models to make a decision at each node in the tree. The purpose of this supplementary file is to provide complete documentation of the model, including the markers and coefficients/weights used in all of the logistic regression models. This documentation is adequate to allow any other investigator to apply exactly the same model to his or her own data sets.

2 Loading the model

We start with a clean R workspace:

```
> ls()
```

```
character(0)
```

Now we load the model that was constructed previously.

```
> f <- "bryanTree.Rda"
> if (file.exists(f)) {
+     load(f)
+ } else {
+     Stangle("BreastCancer08.Rnw")
+     source("BreastCancer08.R")
+ }
> rm(f)
```

The workspace now only contains the pieces needed to compute the model:

```
> ls()
```

```
[1] "bryanMod1" "bryanMod2" "bryanMod3" "bryanMod4" "bryanMod5" "bryanTree"
```

3 Documenting the model

To describe the model, it is necessary to understand that it takes the form of a decision tree, as illustrated in Figure 1. At each node in the tree, we use the values from the RPPA measurements of certain proteins as the features in a logistic regression model. These models involve computing a linear combination of the expression values to produce a score. If the score is positive, you take the left branch in the tree. If the score is negative, you take the right beanch in the tree.

```
> sixcol <- c("red", "orange", "green", "cyan", "blue", "magenta")
> x <- c(6, 3, 9, 11, 13)
> y <- c(10, 8, 8, 6, 4)
> w <- c(3, 2, 2, 2, 2)
> dat <- data.frame(x, y, w)
> lx <- c(5, 1, 11, 9, 15, 7)
> ly <- c(6, 6, 2, 4, 2, 6)
> jam <- function(x, y, w) {
+     lines(c(x, x), c(y, y - 1))
+     lines(c(x - w, x + w), c(y - 1, y - 1))
+     lines(c(x - w, x - w), c(y - 1, y - 2))
+     lines(c(x + w, x + w), c(y - 1, y - 2))
+     text(x - w/2, y - 0.7, ">0")
+     text(x + w/2, y - 0.7, "<0")
+ }
```

For example, the model at the first (highest) node, is as follows:

```
> bryanMod1

Call:  glm(formula = group ~ ER + GATA3.BD + PR + HER2 + HER2p1248 +      CCNE1, family = binomial, data

Coefficients:
(Intercept)           ER     GATA3.BD           PR         HER2    HER2p1248
    -1.6765       1.3281       2.0095       0.6018      -1.1739      -0.3942
      CCNE1
     0.7282

Degrees of Freedom: 711 Total (i.e. Null);  705 Residual
Null Deviance:           965.3
Residual Deviance: 190.9        AIC: 204.9
```

From the "call", we see that six proteins are included in the model. From the coefficients, we see that the score is computed from the formula

$$\texttt{score} = -1.6765 + 1.3281 * \texttt{ER} + 2.0095 * \texttt{GATA3.BD} + 0.6018 * \texttt{PR} - 1.1739 * \texttt{HER2} - 0.3942 * \texttt{HER2p1248} + 0.7282 * \texttt{CCNE1}.$$

When the expression levels of `HER2` and/or `HER2p48` are high enough, the score will be negative, and the sample will be assigned to one of the groups in the right half of Figure 1. If instead the levels of `ER`, `PR`, `GATA3.BD` or `CCNE1` are high enough, then the score will be positive and we will proceed to Node 2 to decide whether to put these samples into group 1 or into group 2.

Here are the cofficients of the formulas at the rest of the nodes in the decision tree.

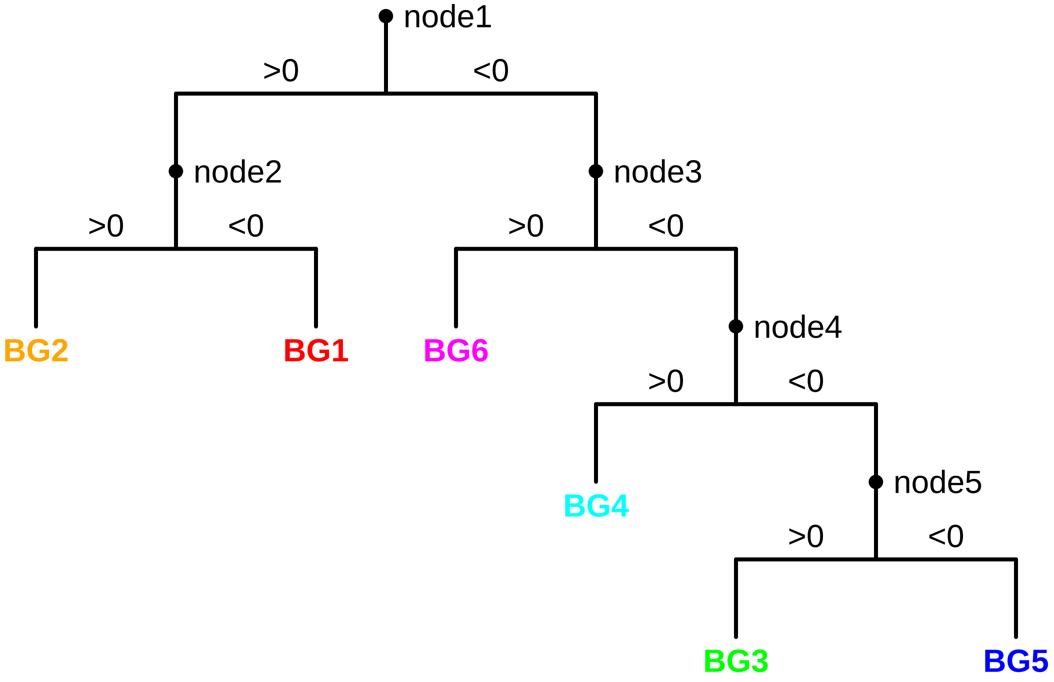

Figure 1: Decision tree for predicting which patients belong to which of the six classes of breast cancer revealed by semi-supervised clustering with ten chosen markers.

3.1 Node 2

```
> bryanMod2

Call:  glm(formula = group ~ PR + HER2p1248 + ER + Bcl2 + GATA3.BD +      CCNB1, family = binomial, data

Coefficients:
(Intercept)           PR    HER2p1248           ER         Bcl2     GATA3.BD
     1.7020       3.9584       0.9469      -2.1627       0.8912      -1.4020
      CCNB1
    -1.1137

Degrees of Freedom: 293 Total (i.e. Null);  287 Residual
Null Deviance:           407.1
Residual Deviance: 63.73         AIC: 77.73
```

3.2 Node 3

```
> bryanMod3

Call:  glm(formula = group ~ HER2p1248 + HER2, family = binomial, data = tdata)

Coefficients:
(Intercept)    HER2p1248         HER2
     -4.584        2.896        1.505

Degrees of Freedom: 417 Total (i.e. Null);  415 Residual
Null Deviance:           469
Residual Deviance: 50.55         AIC: 56.55
```

Note that node 3 separates group 6 (which consists primarily of HER2 positive patient tumors) from nodes 3, 4, and 5 (which consist primarily of trimple negative tumors or hormone receptor positive tumors), so the automated selection of antibodies for (total or phosphorylated) `HER2` is not surprising.

3.3 Node 4

```
> bryanMod4

Call:  glm(formula = group ~ CCNB1 + CCNE1, family = binomial, data = tdata)

Coefficients:
(Intercept)        CCNB1        CCNE1
     -3.091        3.139        2.399

Degrees of Freedom: 313 Total (i.e. Null);  311 Residual
Null Deviance:           400.2
Residual Deviance: 57.91         AIC: 63.91
```

Interestingly, node 4 is distinguished by the cell cycle genes `CCNB1` and `CCNE1`, and so probably reflects a distinction between subtypes based on proliferation.

3.4 Node 5

```
> bryanMod5

Call:  glm(formula = group ~ ER + PR + GATA3.BD, family = binomial,      data = tdata)

Coefficients:
(Intercept)           ER           PR     GATA3.BD
      3.044        3.337        2.026        1.748

Degrees of Freedom: 208 Total (i.e. Null);  205 Residual
Null Deviance:            240.9
Residual Deviance: 58.65         AIC: 66.65
```

Node 5 distinguishes the (primarily) hormone receptor positive tumors in group 3 from the (primarily) triple negative tumors in group 5.

4 Using the model

The actual implementtion of the logic of the decision tree structure is coded in the `bryanTree` function:

```
> bryanTree

function (newdata)
{
    p1 <- predict(bryanMod1, newdata = newdata)
    p2 <- predict(bryanMod2, newdata = newdata)
    p3 <- predict(bryanMod3, newdata = newdata)
    p4 <- predict(bryanMod4, newdata = newdata)
    p5 <- predict(bryanMod5, newdata = newdata)
    result <- rep(NA, nrow(newdata))
    result[p1 > 0 & p2 > 0] <- "BG2"
    result[p1 > 0 & p2 <= 0] <- "BG1"
    result[p1 <= 0 & p3 > 0] <- "BG6"
    result[p1 <= 0 & p3 <= 0 & p4 > 0] <- "BG4"
    result[p1 <= 0 & p3 <= 0 & p4 <= 0 & p5 > 0] <- "BG3"
    result[p1 <= 0 & p3 <= 0 & p4 <= 0 & p5 <= 0] <- "BG5"
    result
}
```

In order to apply the model, you need a data frame that includes a column for each of the protein markers used in the logiastic regression formulas at the various nodes of the decision tree. You then simply pass that data frame into the `bryanTree` function, and get back a character vector where each entry gives the group to which the correpsonding sample has been assigned.

To illustrate, we are going to go through a rather silly exercise of generating some random data. First, we collect a comprehensive list of all the proteins used in the steps of the model.

```
> markerSet <- sort(unique(unlist(lapply(1:5, function(i) {
+     x <- eval(as.name(paste("bryanMod", i, sep = "")))
+     attr(terms(x), "term.labels")
```

```
+ }))))
> markerSet
```

```
[1] "Bcl2"      "CCNB1"     "CCNE1"     "ER"        "GATA3.BD"  "HER2"      "HER2p1248"
[8] "PR"
```

Now we generate some random data:

```
> N <- length(markerSet)
> M <- 1000
> sillyData <- as.data.frame(matrix(rnorm(N * M), nrow = M, ncol = N))
> colnames(sillyData) <- markerSet
```

Now we apply the classifier and peek at the results:

```
> predictions <- bryanTree(sillyData)
> predictions[1:10]
```

```
 [1] "BG5" "BG6" "BG3" "BG3" "BG2" "BG4" "BG3" "BG3" "BG4" "BG1"
```

```
> table(predictions)
```

```
predictions
BG1 BG2 BG3 BG4 BG5 BG6
143 143 350 114 176  74
```

5 Appendix

```
> sessionInfo()
```

```
R version 2.8.1 (2008-12-22)
i386-pc-mingw32

locale:
LC_COLLATE=English_United States.1252;LC_CTYPE=English_United States.1252;LC_MONETARY=English_United Sta

attached base packages:
[1] stats     graphics  grDevices utils     datasets  methods   base
```
